# Application of Hyperspectral Imaging and Deep Learning for Robust Prediction of Sugar and pH Levels in Wine Grape Berries

**DOI:** 10.3390/s21103459

**Published:** 2021-05-15

**Authors:** Véronique Gomes, Ana Mendes-Ferreira, Pedro Melo-Pinto

**Affiliations:** 1 CITAB—Centre for the Research and Technology of Agro-Environmental and Biological Sciences, Inov4Agro—Institute for Innovation, Capacity Building and Sustainability of Agri-Food Production, Universidade de Trás-os-Montes e Alto Douro, 5000-801 Vila Real, Portugal; veroniquegomes@gmail.com (V.G.); anamf@utad.pt (A.M.-F.); 2 WM&B—Laboratory of Wine Microbiology & Biotechnology, Department of Biology and Environment, Universidade de Trás-os-Montes e Alto Douro, 5000-801 Vila Real, Portugal; 3 BioISI—Biosystems & Integrative Sciences Institute, Faculty of Sciences, University of Lisbon, Campo Grande, 1749-016 Lisbon, Portugal; 4 Departamento de Engenharias, Escola de Ciências e Tecnologia, Universidade de Trás-os-Montes e Alto Douro, 5000-801 Vila Real, Portugal

**Keywords:** machine learning, convolutional neural networks, transfer learning, hyperspectral imaging, prediction, grape berries

## Abstract

Remote sensing technology, such as hyperspectral imaging, in combination with machine learning algorithms, has emerged as a viable tool for rapid and nondestructive assessment of wine grape ripeness. However, the differences in terroir, together with the climatic variations and the variability exhibited by different grape varieties, have a considerable impact on the grape ripening stages within a vintage and between vintages and, consequently, on the robustness of the predictive models. To address this challenge, we present a novel one-dimensional convolutional neural network architecture-based model for the prediction of sugar content and pH, using reflectance hyperspectral data from different vintages. We aimed to evaluate the model’s generalization capacity for different varieties and for a different vintage not employed in the training process, using independent test sets. A transfer learning mechanism, based on the proposed convolutional neural network, was also used to evaluate improvements in the model’s generalization. Overall, the results for generalization ability showed a very good performance with RMSEP values of 1.118 °Brix and 1.085 °Brix for sugar content and 0.199 and 0.183 for pH, for test sets using different varieties and a different vintage, respectively, improving and updating the current state of the art.

## 1. Introduction

The science of winemaking has significantly evolved at every stage of its production process, starting at the vineyard where much is defined about the quality of wine. In addition to phytosanitary status, the evaluation of grape quality is mainly associated with grape ripeness, based on the evolution of enological parameters over time, which determines the optimal time for harvesting depending on the desired wine to be obtained. Monitoring maturation faces problems related to the huge variability of grape composition, grape variety, and terroir. Usually, this evaluation is done through classic physical and chemical methods performed offline, using a limited number of samples, which are time-consuming, costly, and invasive and which generate chemical waste. In recent years, extensive studies by different academic researchers and producers have been conducted, aiming the development of innovative and less expensive approaches in order to accomplish a faster, nondestructive, noninvasive, and ultimately more sustainable grape maturity assessment. In this context, hyperspectral imaging in reflectance mode [1,2,3,4,5,6,7,8,9,10,11,12,13,14,15,16] has proven to be a viable alternative to classic techniques in the determination of enological parameters that are important for ripeness assessment and harvest date decision. This imaging technology integrates spectroscopy and digital imaging techniques and allows collecting information about the intensity of light reflected by grapes as a function of their wavelength [3,17,18]. Additionally, hyperspectral imaging allows the acquisition of a large number of samples to assess grape ripeness locally in the vineyard, being of important added value for the industry.

Together with the help of effective machine learning methods, hyperspectral imaging allows estimating multiple enological parameters from the same spectral data, since the reflected light depends on the chemical composition of grapes. In fact, due to the complex spatial wavelength structure, which contains information about all chemical compounds present in grapes and with their overlapping peaks, it is imperative to use learning algorithms to convert the spectral data into the desired enological information. These methods have the ability to generate learning models from training samples composed of measured spectra and the measured enological information of the samples. Thus, once the model is established, they are able to predict the chemical information of interest for new sets of samples. Today, the methods commonly applied for predicting enological parameters of grape berries from spectral data include partial least squares (PLS) [1,4,5,6,7,10,11,12,13,16,19,20], support vector machine (SVM) [2,8], and artificial neural networks (ANNs) [3,4,15,21,22]. However, there are some drawbacks in these methods, such as the dimensionality effect (difficulties in processing the complete spectrum, requiring dimensionality reduction processes). Therefore, the use of deep learning methods, which are emerging in computer science with excellent results in extracting complex patterns of data for a wide field of applications, can be a plus in this prediction context. Deep learning is a class of machine learning algorithms that are most often used for classification, but also employed in regression problems, with successful applications in object classification, object detection, and facial expression recognition, among others. Within deep learning, convolutional neural networks (CNNs) are one of the most popular architectures, showing great potential with innovative results in a variety of fields dealing with images, including remote sensing image classification [23,24,25,26]. Despite being mainly applied to classify two-dimensional (2D) images [27], CNNs have also been used to analyze one-dimensional (1D) data, such as speech recognition, text classification, and, more recently, spectral analysis. However, there are only a few recent studies showing that one-dimensional convolutional neural networks (1D CNNs) can be successfully applied to spectroscopic measurements (1D) in both classification [28,29,30,31,32] and regression [33,34,35,36,37,38,39] problems.

The present work reports the building of one-dimensional convolutional neural networks toward the assessment of grape ripeness focusing on sugar content and pH, two essential maturity indices. Moreover, the broad goal of this study was to assess the behavior and performance of 1D CNNs, addressing the issue of natural variability, in the following relevant tasks: (i) effect of different preprocessing spectral data methods on samples from the same variety for different vintages in the model training, validation, and testing; (ii) generalization ability of the best 1D CNN from the previous task using as independent test set new varieties not employed during the training step; (iii) generalization ability for the same variety but using a different vintage, i.e., using, as an independent test set, samples from a different vintage not employed in the model creation (training and validation). Considering the large variability within vintages/varieties and between vintages/varieties, derived from different conditions such as climate variation, sun exposition, water availability, soil quality, and altitude, inspecting the generalization ability of the 1D CNN with different vintage/varieties is fundamental to acquire robustness of the final methodology. If it is not possible to reach the task of generalization in grapes, then the solution becomes less attractive from the industry point of view, since it will be necessary to fully retrain the model annually to be used for that particular year [3,4]. The scientific literature for this generalization aspect is practically nonexistent, and only a few works training models with grapes from one vintage and testing them with grapes from another vintage can be found [3,4,8,13,19]. Regarding task (i), although deep learning can be trained on datasets without the use of preprocessing [40], there have been some recent works published in the scientific literature where the use of preprocessing combined with deep learning was applied to spectroscopic data, leading to results improvement. For instance, in [37], the raw spectrum was standardized using the standard normal variate (SNV) method before being fed into the CNN model; [39] evaluated the effect of using the original spectra or of the spectra preprocessed by the multiplicative scatter correction (MSC) method in a 1D CNN model for the prediction of corn seed, showing an improvement in accuracy for MSC + 1D CNN; [28] used a preprocessing strategy, combining different spectral preprocessing techniques, to develop CNN models in different spectroscopic datasets; [38] implemented extended multiplicative scatter correction (EMSC); [31] applied a Savitzky–Golay (SG) filter and logarithm methods to the reflectance spectra before 1D CNN.

To the authors’ best knowledge, this work is innovative in the use of 1D CNN to predict enological parameters in whole grapes using hyperspectral imaging in reflectance mode. In addition, our 1D CNN architecture was designed to make predictions, unlike most CNNs applications which are developed for classification problems, and its relevant hyperparameters were optimized through Bayesian optimization with a Gaussian process.

## 2. Material and Methods

### 2.1. Data Acquisition

Three native Portuguese varieties that are widely used to produce port wine were selected due to their high relevance for Symington Family Estates, our industrial partner and one of the largest and most important port wine producers in the world. Grape samples of the three varieties considered, Touriga Franca (TF), Touriga Nacional (TN), and Tinta Barroca (TB), were harvested from the vineyards of Quinta do Bomfim, Pinhão, Portugal. TF samples were collected in 2012, 2013, 2014, 2016, 2017, and 2018, while TN and TB samples were harvested in 2013, 2014, 2016, and 2017 (see Table 1). For each vintage/variety set, the grape berries were collected between the beginning of veraison and maturity, from three different locations inside the vineyard (from vine trees with small, medium, and large vigor), assembling a total of 1748, 454, and 463 grape samples for TF, TN, and TB, respectively. More details on the characterization of the collected samples per vintage and variety can be found in Section 3.1.

Line-scan hyperspectral image acquisition was performed in our laboratory-based imaging system using fresh grape samples. Each sample comprised six or 12 grape berries, randomly collected from a single bunch with their pedicel attached. After imaging, all samples were frozen at −18 °C before determining the analytical enological values. The procedures regarding the experimental setup for hyperspectral imaging acquisition and the computation of reflectance spectrum were previously described by the authors in [3,4,8,15]. Therefore, the reader is directed to additional references for a detailed description. In summary, the hyperspectral data were collected using the following hyperspectral imaging system acquisition: a hyperspectral camera, composed of a JAI Pulnix (JAI, Yokohama, Japan) black-and-white camera and a Specim Imspector V10E spectrograph (Specim, Oulu, Filand); lighting, using a lamp holder with 300 × 300 × 175 mm^3^ (length × width × height) that held four 20 W, 12 V halogen lamps and two 40 W, 220 V blue reflector lamps (Spotline, Philips, Eindhoven, the Netherlands), powered by continuous current power supplies to avoid light flickering at only 110 V to reduce lighting and prevent camera saturation. The distance between the camera and the sample base was 420 mm, and the camera was controlled with Coyote software from JAI. After imaging, the grape berries were identified, and their data were extracted using a threshold-base segmentation method. Furthermore, reflectance was used to correct signal variations caused by the illumination and the hyperspectral camera. This step was performed by recording the dark current signal (*DI*) associated with the hyperspectral camera output, acquired with the camera shutter closed (0% reflectance), and the intensity of light that illuminated the grape berries (*SI*), using a white reference target, Spectralon (Specim, Oulu, Filand), which reflects almost all the light reaching its surface in the ultraviolet, visible, and infrared wavelengths. Thus, for a given wavelength, *λ*, and position, *x*, the reflectance, *R*, was computed as follows:

(1) $$R\left(x,\lambda \right)=\frac{GI\left(x,\lambda \right)-DI\left(x,\lambda \right)}{SI\left(x,\lambda \right)-DI\left(x,\lambda \right)},$$

where *GI* is the intensity of light reflected by the grapes.

The reference values of sugar content and pH that allowed building and evaluating the prediction models were determined by conventional chemical analysis. Thus, the grapes were defrosted and crushed, and then the sugar content (measured in °Brix) and pH were analyzed by refractometry, using a handheld refractometer (ATAGO N1, ATAGO CO., Ltd., Tokyo, Japan), and by potentiometry, using an automatic titrator (Crison micropH 2002, Crison, Barcelona, Spain), respectively, according to validated standard methods [41].

After data acquisition, each acquired spectrum was paired with the sugar and pH reference values to assemble the final datasets.

### 2.2. Spectral Preprocessing

In order to evaluate the effect of reflectance spectrum preprocessing on the predictive model, three well-known techniques were considered: multiplicative scatter correction (MSC), min–max normalization (Norm), and Savitzky–Golay (SG). The use of preprocessing techniques is an often-used step in spectroscopic measurements to minimize/eliminate fluctuations in the measured light intensities, which for the present purpose are due to the grape berry size and curvature [3,4,15]. In addition, this process is usually important for the development of regression-type algorithms, since they generally benefit from better-conditioned data.

The MSC technique is probably the most widely used transformation technique in visible/NIR spectroscopy that aims to correct the scatter level of each spectrum in such a way that all samples appear to have the same level as the reference spectrum [42]. The scatter correction is achieved by regressing each spectrum against the reference spectrum (usually the mean of the training set), and then correcting the recorded spectrum using the slope and intercept of the linear fit [43]. For the min–max normalization approach, each spectrum is normalized into a 0–1 range. First, the minimum and maximum values of all the intensities of a given spectrum are computed, and then the normalized spectrum is obtained through subtracting the minimum value from the given spectrum and dividing by the range (maximum–minimum). The Savitzky–Golay method is a moving-window-based local polynomial least-square fitting procedure, being one of the most commonly employed smoothing and differentiation techniques [44]. There are two important parameters that must be taken into account when the Savitzky–Golay technique is used: the window size and the order of the polynomial. The window size specifies the number of data points that will be used to fit a polynomial regression model of a given order. The choice of too small a window may not be enough to reduce the noise. On the other hand, the choice of too large a window might filter relevant information and misrepresent the spectra [45]. The second parameter specifies the degree of the polynomial used during the fitting and conditions the highest derivative that can be estimated. Usually, the second-order polynomial is employed, and the most used window sizes range between seven and 15 points. In this work, the Savitzky–Golay technique was employed to perform both smoothing and differentiation, allowing an estimation of the derivatives of the smoothed signal. This was done by inferring the first-order derivative from a best local least-squares polynomial fit at each wavelength. A second-order polynomial was used with a window size of 15 points.

### 2.3. One-Dimensional Convolutional Neural Network Architecture

**Figure 1:**
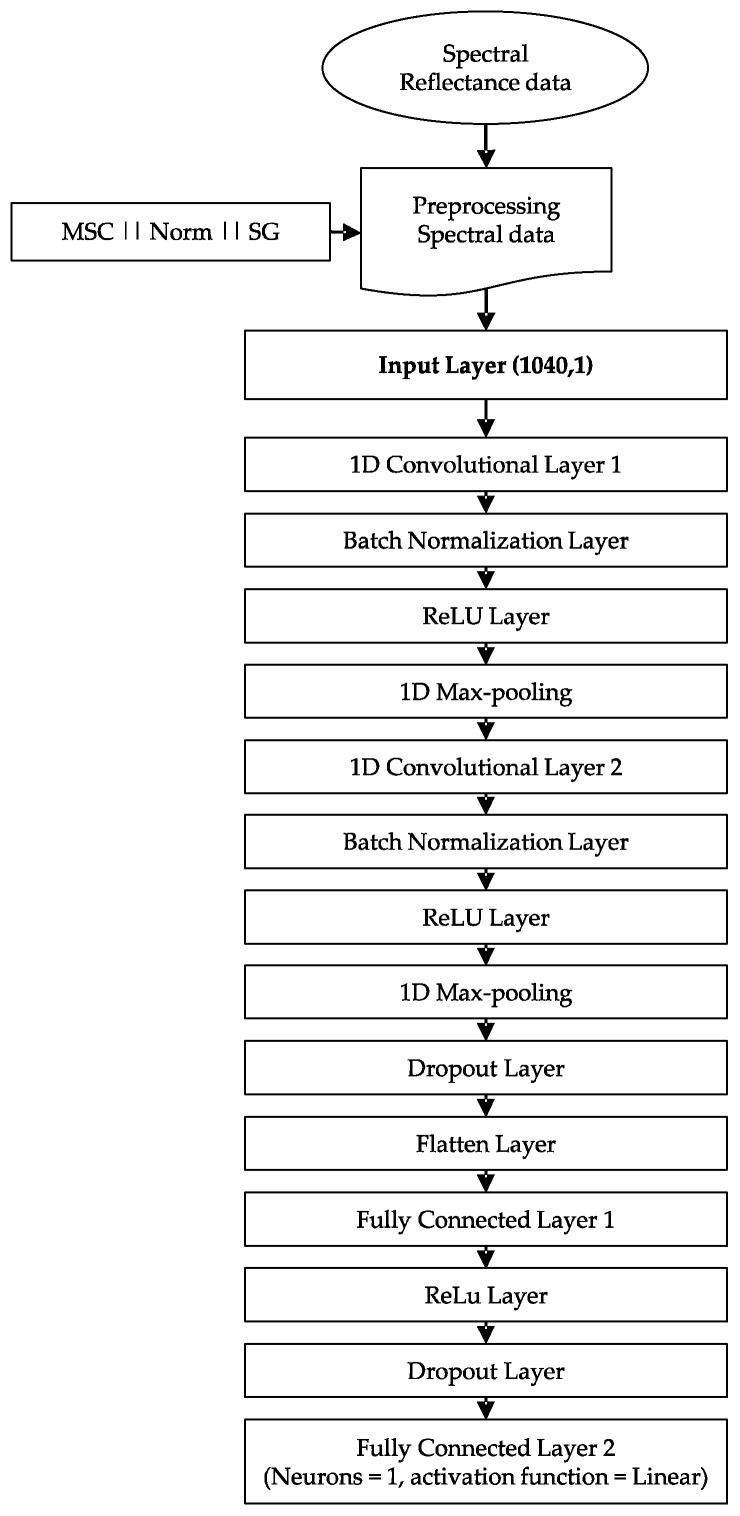
One-dimensional convolutional neural network architecture design.

A one-dimensional convolutional neural network architecture was developed in Python (Python Software Foundation, Wilmington, DE, USA) using KERAS package version 2.2.4 (https://keras.io/ accessed on 25 March 2021). Typically, a CNN architecture involves an input layer, several hidden layers (convolutional layers, polling layers, fully connected layers), and an output layer. The input and output of our 1D CNN were the grape reflectance spectrum (1040 × 1) and the sugar content and pH predictions, respectively. The feature extraction part of our 1D CNN consisted of two one-dimensional convolutional layers (with a stride of 1, the ‘same’ padding, and rectified linear unit (ReLU) activation function). A batch normalization layer was added after each convolution layer in order to accelerate training, as well as provide some regularization in the model [46]. The output of each convolutional layer was passed to a max pooling layer in which the pooling size of the first layer was set to two and that of the second layer was set to five. The last output feature maps from the pooling layer were flattened and fed to a fully connected dense layer. Lastly, the output layer was a single dense neuron with a linear activation function. A dropout layer was added after the last pooling layer and another was added between the fully connected layer and the output layer to avoid overfitting. The weights of the convolutional kernels and fully connected layer were initiated using ‘Glorot uniform’ initialization [47]. The mean squared error (MSE) was defined as the loss function, and early stop was used. Furthermore, the training process was done using the Adadelta optimizer [48] and was stopped at the number of epochs with the lowest mean square error for validation patterns. The detailed 1D CNN architecture proposed in this work is shown in Figure 1.

### 2.4. Hyperparameter Optimization

The successful implementation of deep learning approaches strongly depends on the set of hyperparameters used during model training. Unfortunately, the relationship between hyperparameters and the performance of the model is still unclear, making it difficult to calculate the value of each hyperparameter to be used in the model [49]. In this context, hyperparameter optimization is required and, despite being a challenging and time-consuming approach, it is a key issue in deep learning algorithms. The optimization of hyperparameters can be performed through manual or automatic search methods. In practice, manual search consists of training a number of models with different combination of hyperparameter values manually set in which the best-performing model is chosen. Nevertheless, it requires the user to have background knowledge and practical experience with the particularities of the data, hindering its application by nonexpert users [49]. To overcome this issue, automatic hyperparameter optimization approaches such as Bayesian optimization have been widely used in recent years [38,49,50,51,52,53,54,55]. In this work, Bayesian optimization with a Gaussian process (BOGP) was used to tune a set of hyperparameters (see Table 2). BOGP has become popular in the literature due to its ability to model the objective function, as well as the uncertainty associated with predictions. The algorithm relies on building a probabilistic model (using the Gaussian process) of the function, mapping hyperparameter values to the objective function evaluated on a validation set, updating the probabilistic model based on new evaluations, and then exploiting this model to decide the next hyperparameter values to evaluate in the true objective function. An acquisition function [50] is used to evaluate the “goodness” of candidate points in the true objective function. In particular, the Gaussian process selects the next hyperparameter values by finding the maximum of the acquisition function; then, the model is updated to take the new data into account. At each iteration, the model is progressively refined. The final aim is to find the input hyperparameter values that provide the best possible output value.

The algorithm was implemented in Python using the package scikit-optimize version 0.7.4 (https://scikit-optimize.github.io accessed on 25 March 2021). The optimization was initialized using 20 random hyperparameter sets followed by up to 200 iterations of the Gaussian process, using the expected improvement (EI) as the acquisition function [49]. In each iteration, a new 1D CNN was built from the hyperparameter set selected and trained according to the architecture specified in Section 2.3. Final training of the 1D CNN was done with the identified best hyperparameter values.

### 2.5. Model Training, Validation, and Test Methodology

In order to perform each task presented in Section 1, the acquired hyperspectral data were divided into training, validation, and test sets, using a stratified scheme based on the percentiles described below. Tasks (i) and (ii) used the same pair of training and validation sets, differing only in the independent test set.

For task (i), i.e., the effect of different preprocessing spectral data, all samples of Touriga Franca (from 2012 to 2018) were used to create the 1D CNN model using the Bayesian optimization described in Section 2.4. Thus, the reference measurements for each Touriga Franca vintage were grouped into five intervals according to the 20th, 40th, 60th, and 80th percentiles. In each group of percentile intervals, 10% of samples were set aside for the independent test set, another 10% were set aside for the validation set, and the remaining samples were used for the training set. Each final dataset (training, validation, and independent test sets) was formed by collecting the respective TF samples partitioned for each vintage (from 2012 to 2018). The training and validation sets were used to find the best set of hyperparameter values. In addition, the validation set was used to tune the network weights. Lastly, the optimized models were evaluated and compared with the independent test set using the root-mean-square error of predictions (RMSEP) as the evaluation criterion.

Regarding task (ii), i.e., the generalization ability using different varieties, the best 1D CNN models created in task (i) for sugar and pH parameters were fed without training with a new independent test set composed of TB and TN varieties of all years (2013 to 2017).

Lastly, concerning task (iii), i.e., the generalization ability using different vintage, samples of TF from 2012 to 2017 vintages were used for training. The final training and validation sets were created using the stratified scheme based on percentiles, following the same procedure for task (i). Bayesian optimization with a Gaussian process was also used to find the best set of hyperparameters and to create the final model. The generalization ability of the established 1D CNN model was evaluated using all samples from TF 2018 vintage as an independent test set.

The root-mean-squared error of prediction (RMSEP) for the independent test set was used to assess the generalization capacity of the models.

## 3. Results

### 3.1. Sampling Characterization

**Figure 2:**
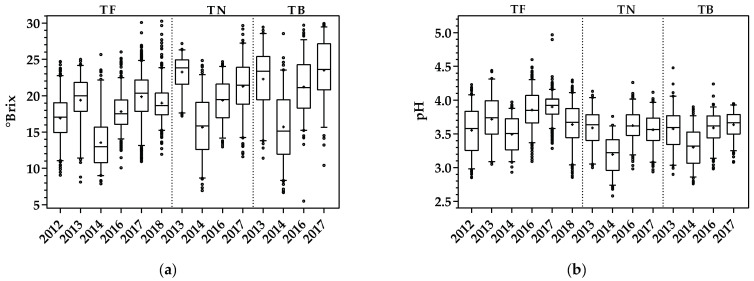
Sampling characterization for each vintage and variety: (**a**) sugar reference measurements; (**b**) pH reference measurements.

The boxplots of reference measurements obtained for sugar content and pH by the conventional analytical techniques are presented in Figure 2. The boxes represent the 25th, 50th, and 75th percentiles, the whiskers represent the fifth and 95th percentiles, the lower and upper open circles represent the minimum and maximum values, respectively, and the plus symbol denotes the mean values. These enological values were used as reference values to create and test the proposed models. From Figure 2, it is possible to verify the difference between grape varieties within a vintage and between vintages for each grape variety, which complicates the prediction for new vintages and/or varieties.

Regarding sugar content, TB presented a 95th percentile much larger than TF and TN for most vintages, with the exception of 2014, which presented slightly higher values than TN and TF varieties. The same can be verified for the fifth percentile, with the exception of 2014 samples (slightly lower than TF and TN) and TN 2013 samples (which showed a much larger fifth percentile, with a similar value to the 25th percentile of TF 2013). The amplitude of the box representing the 25th, 50th, and 75th percentiles was also larger for TB and TN. Overall, within varieties and all vintages, TF values ranged from 7.87 to 30.26 °Brix, while, in TN and TB, the minimum values were 6.95 and 5.48 °Brix and the maximum values were 29.66 and 29.95 °Brix, respectively.

Concerning pH, TN and TB displayed lower values for the 95th and fifth percentiles when compared with TF, except for the 2013 vintage that showed similar values for the fifth percentile. Within varieties and considering all samples, TF values of pH ranged from 2.85 to 4.97, TN values varied from 2.58 to 4.26, and TB values ranged between 2.76 and 4.48. For more information regarding the descriptive statistics of the datasets used in the subsections below, see the boxplots presented in Appendix A.

### 3.2. Effect of Spectral Preprocessing in 1D CNN Model

The best hyperparameters achieved through Bayesian optimization with a Gaussian process are presented in Table 3. The difference obtained between the best sets of hyperparameters for different preprocessing techniques can be explained by the difference in the input spectra (after preprocessing) resulting in different extracted features. We can also observe from Table 3 that BOGP selected the same number of neurons for the FCN (it did not depend upon the extracted features but on the feature vector dimension) and the same batch size for the different preprocessing techniques (the regularization of the learning process relied mainly in the input dimension and network architecture). Regarding the number of filters, kernel size, and dropout and learning rates, the best values selected varied for each technique. The best configuration achieved was the same for sugar content and pH data.

Table 4 shows the results obtained for the validation and test sets, using the resulting 1D CNN for each preprocessing method. The low RMSE values in the independent test set demonstrate the good predictive capacity of the developed models using each preprocessing technique, with Savitzky–Golay being the best preprocessing technique for both enological parameters, presenting RMSEP values of 0.755 °Brix and 0.110 for sugar and pH, respectively. In addition, the use of SG preprocessing resulted in the need for fewer epochs (120) to train the model (SG + 1D CNN) than the use of the other two techniques (230 epochs for MSC and 200 epochs for min–max normalization). The best hyperparameter configuration achieved for SG preprocessing was also used with MSC and min–max normalization, confirming that the use of these preprocessing techniques led to worse results with RMSEP values of 1.058 °Brix and 0.164 for MSC and 1.019 °Brix and 0.156 for min–max normalization in sugar content and pH, respectively.

**Figure 3:**
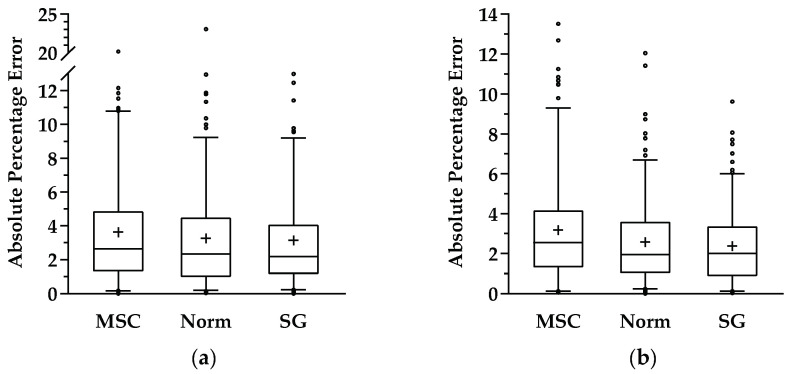
Absolute percentage error of each preprocessing technique regarding the 1D CNN created when applied to independent test samples for (**a**) sugar measurements and (**b**) pH measurements.

Figure 3 illustrates the boxplots of the absolute percentage errors (APE) for the independent test set prediction and for each preprocessing technique, concerning each enological parameter. Regarding the sugar content predictions, the 95th percentiles for SG and min–max normalization were similar and smaller than 9.1%, and the 75th percentiles were smaller than 4.5%, denoting the good predictive capacity of both techniques in combination with the proposed 1D CNN model. On the other hand, the MSC technique seemed to present a slightly inferior performance when compared with the other two, showing 95th and 75th percentiles of APE smaller than 11% and 5%. For pH, the 95th percentile was higher for the MSC technique with an APE value of 9.1%, and better for Norm and SG, with APE values slighter than 7% and 6%, respectively. Concerning the 75th percentiles, MSC presented APE values of 4.1%, while Norm and SG techniques presented APE values similar to or smaller than 3.5%.

**Figure 4:**
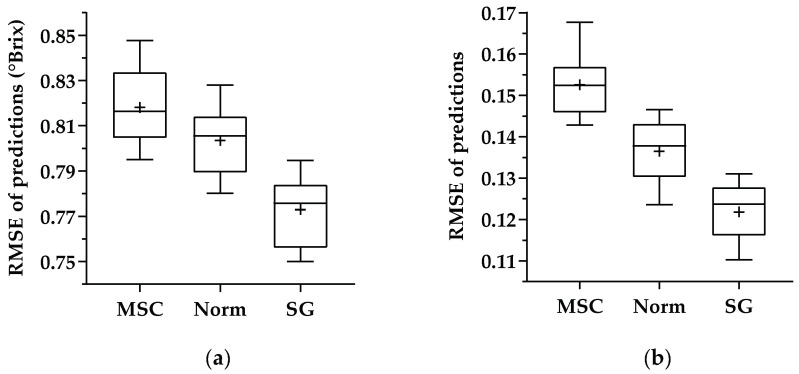
RMSEs of predictions for each preprocessing technique regarding the 1D CNN created and then applied to test samples for (**a**) sugar measurements and (**b**) pH measurements. The boxes represent the 25th, 50th, and 75th percentiles, the whiskers represent the minimum and maximum values, and the plus symbol denotes the mean reference values.

Nevertheless, in order to verify that the good performance presented in Table 4 was not a question of the “good” splitting of that dataset, another 10 random training, validation, and test sets (using the same stratified scheme as before) were created and used to evaluate the performance of the 1D CNN model (Figure 4.). It is clear from the results illustrated in Figure 4 that SG preprocessing presented better results for the prediction of both parameters—sugar content and pH. Consequently, Savitzky–Golay preprocessing was used as the preprocessing method for the remaining tasks.

### 3.3. Generalization Ability: Testing with Different Varieties

**Figure 5:**
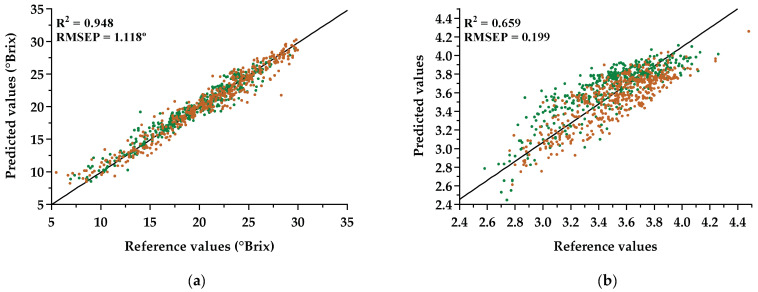
Prediction results of the independent test set with samples of the varieties TN (green points) and TB (orange points) when introduced into the 1D CNN model created with TF samples, regarding (**a**) sugar measurements and (**b**) pH measurements.

For the model generalization ability assessment, the 1D CNN with the spectral data after preprocessing with the SG technique was employed, and an independent test set with the hyperspectral data from TB and TN varieties was used. Figure 5 shows the prediction results obtained for sugar content and pH parameters. These results in terms of RMSEP for the independent test set were 1.118 °Brix and 0.199 for sugar content and pH, respectively.

**Figure 6:**
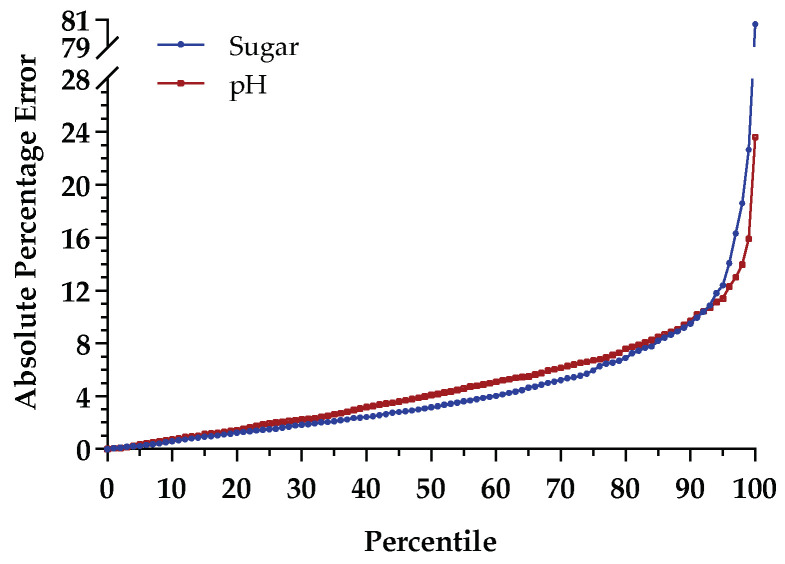
Percentiles for absolute percentage error of sugar and pH in the independent test set (TB and TN).

Figure 6 shows the percentile curves for the absolute percentage errors, regarding sugar (blue curve) and pH (red curve) values. One can notice that the APE values for 95th percentiles in sugar and pH were approximately 12.4% and 11.4%, respectively, while the APE values for the 75th percentile were smaller than 6% for sugar content and smaller than 6.7% for pH. Furthermore, in order to characterize the performance for each variety, a summary of the root-mean-square errors of predictions obtained is presented in Table 5.

### 3.4. Generalization Ability: Testing with a Different Vintage

As mentioned in Section 2.5, samples of TF from 2012 to 2017 were used to train and validate the proposed 1D CNN (TF Model (2012–2017)), while samples from TF 2018 (TF Test (2018)) were employed in order to evaluate the generalization ability regarding a (different) vintage not employed during the training process. The Savitzky–Golay first derivative was applied to the spectra as the preprocessing technique. The best hyperparameters obtained through BOGP optimization are summarized in Table 6.

In Table 7 the predicted results for each enological parameter are shown. The trained 1D CNN, using all samples of TF except for the 2018 vintage, presented a RMSEVs of 1.227 °Brix and 0.182 for sugar content and pH, respectively. For the independent test set, the obtained results in terms of RMSEP were 1.396 °Brix for sugar content and 0.223 for pH.

**Figure 7:**
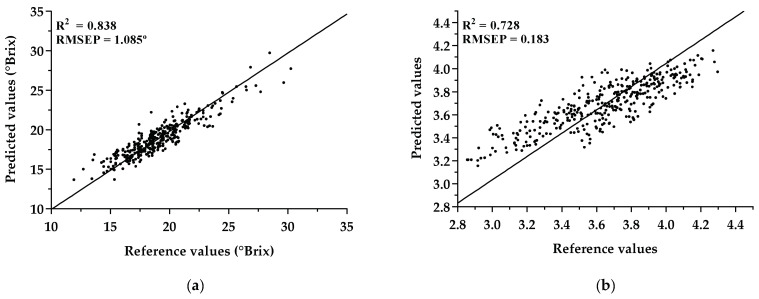
Prediction results of the TL-TF Model (2017) for (**a**) sugar measurements and (**b**) pH measurements.

The authors decided to verify if any improvements could be obtained using a transfer learning mechanism (TL) and carried out an experiment using samples of TF from 2012 to 2016 to pretrain a 1D CNN model (TF Model (2012–2016)). The weights obtained were then used as a weight initialization scheme for training a new model with samples of TF 2017 (TL-TF Model (2017)), corresponding to the fine-tuning of the weights. Visualization of the prediction’s performance for the independent test set, TF Test (2018), obtained for the TL-TF Model (2017), is shown in Figure 7. Here, the new 1D CNN presented an RMSEP of 1.085 °Brix for sugar content and 0.183 for pH, evidencing the improvement of the methodology using a pretrained model.

**Figure 8:**
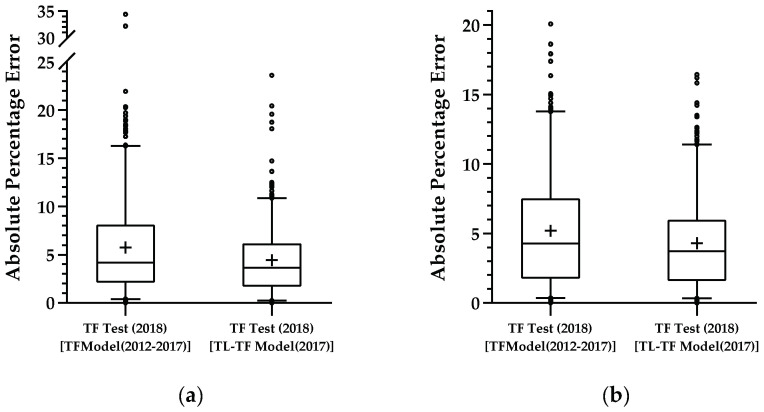
Absolute percentage error of each TF Test 2018 when applied to both created models: (**a**) sugar measurements; (**b**) pH measurements.

In Figure 8, it is possible to see the boxplots of the absolute percentage errors for the independent test set TF Test (2018) when employed in the created TF Model (2012–2017) and in the new TL-TF Model (2017). Thus, the 95th percentiles were smaller in the new TL-TF Model (2017) than in the original TF Model (2012–2017), with APEs less than 11.5% for both enological parameters. In addition, the 75th percentiles were around 6% for APEs in both enological parameters. For the test set in the original TF Model (2012–2017), the 95th and 75th percentiles were below 16% and 8% for sugar and below 14% and 8% for pH.

## 4. Discussion

Our study expands existing approaches to predict important enological parameters in order to evaluate the wine grape maturation stage through hyperspectral imaging technology. This is a complex problem with data presenting high variability due to the large differences in terroir, climate, and grape varieties. Overall, the obtained results outperform those published in the literature, demonstrating the effectiveness and robustness of our methodology, particularly in terms of the model’s generalization ability using different varieties and different vintages than those employed in training. This is an important achievement, indicating that it might be not necessary to train models for every single vintage or variety. Furthermore, as the grapes were in various ripening stages and presented different values of enological parameters (due to the large differences mentioned before), our results suggest that the developed deep learning models were able to capture most of the variations presented in the data, unlike the traditional machine learning models applications presented in the literature.

Analyzing the results obtained for the different preprocessing techniques in Section 3.2, it is possible to verify that Savitzky–Golay was the best technique working in combination with the proposed 1D CNN architecture for predicting sugar content and pH, obtaining the lowest root-mean-squared error (Table 4) and best overall performance (Figure 4). Comparing with the existing literature, [9] employed different preprocessing techniques but for the prediction of anthocyanin concentration using a PLS regression model, and they also found a better performance for the SG preprocessing technique. On the other hand, [11] compared two different preprocessing approaches, using PLS, PCR, and MLR models for the prediction of sugar content, and they concluded that performance was case-dependent. Other authors [4] found that the use of derivatives (SG) revealed an improvement in the prediction results, with MSC showing the worst results, whereas some authors found that the preprocessing techniques did not lead to an improvement in results [7,13].

**Figure A1:**
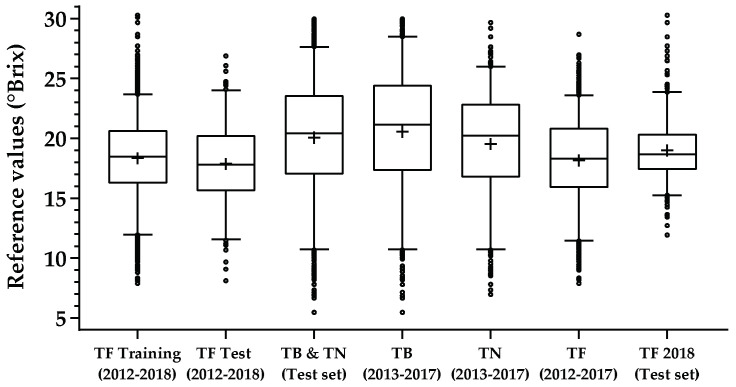
Boxplot of the descriptive statistics for sugar content reference values used for the training process and independent test phases. The boxes represent the 25th, 50th and 75th percentiles, the whiskers represent the fifth and 95th percentiles, the lower and upper open circles represent the minimum and maximum values, and the plus symbol denotes the mean values.

**Figure A2:**
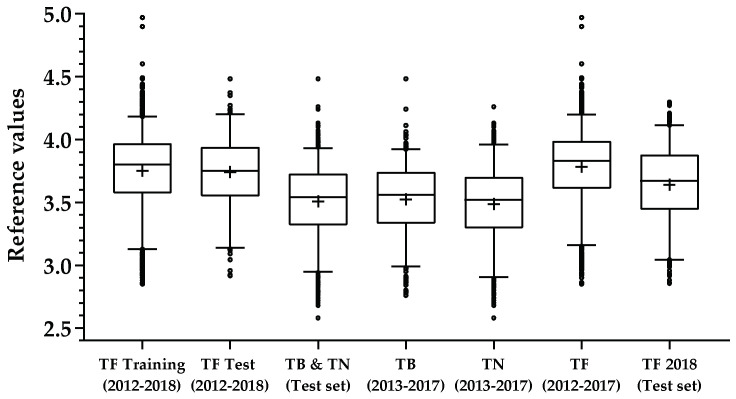
Boxplot of the descriptive statistics for pH reference values used for the training process and independent test phases. The boxes represent the 25th, 50th and 75th percentiles, the whiskers represent the fifth and 95th percentiles, the lower and upper open circles represent the minimum and maximum values, and the plus symbol denotes the mean values.

**Figure A3:**
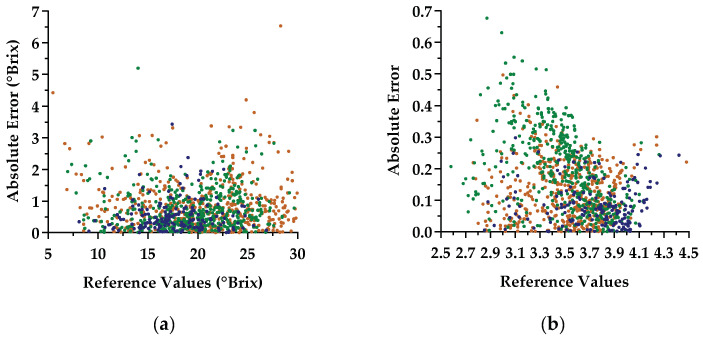
Absolute errors obtained for the independent test set comprising TF (blue point), TB (orange points), and TN (green points) varieties in (**a**) sugar measurements and (**b**) pH measurements.

From the analysis of the results obtained in Section 3.3, in which the model was tested with samples of TB and TN varieties, it is possible to verify an increase from the RMSEP (Table 4) for the TF test set (2012–2018) to the RMSEP (Figure 5) for the test set with other varieties. This increase might be related to the different distributions in sugar content and pH, as shown in Figure A1 and Figure A2 (Appendix A), respectively. Moreover, it is clear that the model performed better for sugar content than for pH, with a minor increase in the RMSEP for the independent test set (TB and TN). In fact, looking at the distribution of the absolute errors of sugar content as a function of the reference values (Figure A3a, Appendix A), one may see that the model could better handle sugar values between 17 and 21 °Brix (25th and 50th percentiles in TB and TN set), which belong to the sample points within the range of values delimited by 25th and 75th percentiles of the TF training set, but it was still able to satisfactorily predict most of the remaining sample points that fell outside the range of values between the 25th and 75th percentiles. This means that, despite the TB and TN test set for sugar content presenting larger variability than the training set (Figure A1, Appendix A), the overall impact on the performance of the created model seems not to be problematic. On the other hand, for pH (Figure A3b, Appendix A), it is clear that the model had a better fit to values larger than 3.5 since the sample points above the 50th percentile for the independent test set fell within the existing values above the 25th percentile for the training set. The increase in RMSE for pH might be related to the distribution of the remaining sample points and to the genetic proximity or distance between varieties, as reported in [8]. Furthermore, this can also be justified by the fact that the acidity seems to be a sensitive case, with small changes in the range of the reference measurements, which may increase the difficulty of the model to learn, thus needing more training samples to capture the patterns present in the data. For the results obtained individually for each variety (Table 5), it can be denoted that the model created with TF (2012–2018) samples generalized better for TN samples and worse for TB samples in terms of sugar content, and the opposite was true for pH. Again, these differences might be justified by the different distributions shown in Figure A1 and Figure A2 (Appendix A), which might result from their different genetic structure along with the differences in terroir, such as temperature, sun exposition, water availability, soil quality, and altitude resulting from the vineyard locations where the samples were collected.

Concerning whether a model created with samples of TF from 2012 to 2017 was able to successfully generalize when a different vintage (TF 2018) was used as independent test set, we observed that, when we built the first model (TF Model (2012–2017)), the results in terms of RMSE were significantly worse (Table 7), which can be related to the fact that fewer samples were used for training, and the 1D CNN was probably unable to properly train the model. However, when a second experiment was done, using a transfer learning mechanism, the results showed a significant improvement (Figure 7) with a drop in RMSE values of approximately 22% for the independent test set. Moreover, the results suggest that the use of pretrained weights can be a plus to improve the performance of our models, indicating that significant improvements can be obtained when using a pretrained model and then fine-tuning such a model with a new small set. This means that we may not need to fully train a new model with all vintages every single year, but only fine-tune a previously trained one with the new vintage.

Concerning our previously published works, in [15], a neural network was trained and tested using TF samples from 2012 which revealed RMSEP values of 0.95 °Brix for sugar content and 0.18 for pH with respective APE values for 95th percentiles of approximately 11%. Moreover, in [3], the model created in [15] was used to test the generalization ability for three independent test sets (each comprising TF, TN, and TB varieties from the 2013 vintage), obtaining RMSEP values of 0.191, 0.170, and 0.176, respectively, and APE values for the 95th percentile larger than 10% for pH. In [4], the study was only conducted using TF 2013 but two approaches (neural networks and PLS) were compared for sugar content prediction, presenting results of 1.350 °Brix and 1.340 °Brix in terms of RMSEP with 86% and 83% of samples having APEs smaller than 10%, respectively, for the NN and PLS approaches. In addition, [8] obtained RMSEP values for sugar and pH of 1.411 °Brix and 0.144, using TF samples from 2012 to 2014 to train and test an SVR algorithm, whereas the model’s generalization capacity was also tested for samples of TN and TB from 2013. The RMSEPs obtained for sugar content were 2.443 and 3.186 °Brix, respectively, while pH presented RMSEP values of 0.253 and 0.303, for the model’s generalization ability. In the present work, in general, the results obtained were better in terms of RMSE and APE, updating our state-of-the art results [3,4,8,15]. Considering other works published in the literature, the results obtained for sugar content in the present work were better than those obtained in [1,2,5,7,10,11]. On the other hand, [12,13] revealed results similar to ours for sugar content but better than ours for pH. However, the authors of those works used homogenates or a larger number of berries per sample compared with the six or 12 berries per sample used in this work, which reduced the problem’s complexity, as well as applicability. As mentioned in [4,15], the use of a larger number of berries reduces the variability in both the acquired spectra and the reference enological values measured, with positive bias in the final results. Herein, the decision to use a small number of grapes offers new possibilities for the selection of the best berries from each bunch to produce specific high-quality wines [3,8]. To highlight the results obtained in the present work, a comparison with other published works in the literature is presented in Table A1 (Appendix A). Most of the published works in Table A1 (Appendix A) only took into account samples from no more than one or two harvested years, except for [5,8] that used three different vintages to create the models. As shown in Section 3.1, the use of more vintages implies larger data variability, which makes it harder to obtain good results using traditional machine learning models. However, deep learning can capture these variations and learn valuable features to make the predictions robust. In addition, another benefit of our 1D CNN over the machine learning methods employed in the literature is its ability to automatically perform feature extraction (through learning), which improves both the model’s accuracy and the model’s generalization ability.

## 5. Conclusions

In this work, we presented a methodology for predicting two important enological parameters through hyperspectral imaging, focusing on the following main issues: the effect of spectral preprocessing and generalization ability for different varieties and for different vintages not employed in training. To do so, a 1D convolutional neural network architecture was designed, and the corresponding hyperparameters were optimized using Bayesian optimization with a Gaussian process. Moreover, a transfer learning mechanism was also employed.

An important feature of this work relies on the robustness and effectiveness of the proposed 1D CNN models that revealed excellent overall performance, even for different varieties or vintages not employed during training, thereby improving and expanding the current state of the art. Regarding the effect of preprocessing techniques, Savitzky–Golay was the most suitable technique in combination with the proposed 1D CNN model.

This study proves that the combination of hyperspectral imaging with appropriate predictive learning methods (e.g., 1D CNN) can be a rapid, nondestructive, and efficient alternative to the conventional analytical techniques, representing an important step toward a more sustainable grape quality assessment.

Nevertheless, we believe that the proposed approach can still improve with the addition of new samples, e.g., including more vintages and/or blending the TF, TB, and TN data into a unique dataset, using a random sampling method to split the data into training, validation, and test sets. Furthermore, an increase in the number of convolution layers or the use of different deep learning models should also be the subject of future research, as well as the identification of important spectral features by studying potential mechanisms of influence for sugar content and pH in the reflectance spectra. Lastly, the novel prediction model presented here is highly suitable for a rapid and nondestructive wine grape ripeness assessment using laboratory-acquired data. It is important for future research to undertake field hyperspectral data acquisition to upscale this model to field conditions.

## Figures and Tables

**Table 1:** Number of samples collected for each vintage and variety.

Vintage	Variety	No. of Samples
2012	Touriga Franca	240
2013	Touriga Franca	81
	Touriga Nacional	60
	Tinta Barroca	82
2014	Touriga Franca	120
	Touriga Nacional	118
	Tinta Barroca	120
2016	Touriga Franca	407
	Touriga Nacional	132
	Tinta Barroca	143
2017	Touriga Franca	540
	Touriga Nacional	144
	Tinta Barroca	118
2018	Touriga Franca	360

**Table 2:** Bayesian optimization hyperparameter settings.

Hyperparameter	Range Values
Convolution layer 1—number of filters (#Filters 1)	5–256
Convolution layer 1—kernel size 1	3–100
Convolution layer 2—number of Filters (#Filters 2)	5–256
Convolution layer 2—kernel size 2	3–100
Dense No. of neurons (neurons)	4–256
Dropout rate (dropout 1/2)	0.1–0.6
Learning rate (LR)	0.01–0.06
Batch size	8–260

**Table 3:** Optimized hyperparameters of 1D CNN for each preprocessing method using BOGP.

Preprocessing	#Filters 1	Kernel Size 1	#Filters 2	Kernel Size 2	Neurons	Dropout 1/2	LR	Batch Size
MSC	39	40	60	7	128	0.20/0.15	0.050	8
Norm	34	50	47	9	128	0.15/0.15	0.039	8
SG	60	50	60	3	128	0.40/0.20	0.033	8

**Table 4:** Model performance of the optimized 1D CNN for each preprocessing method.

Parameter	Preprocessing	Validation Set	Test Set
		RMSEV	RMSEP
Sugar	MSC	0.765 °Brix	0.806 °Brix
	Norm	0.743 °Brix	0.791 °Brix
	SG	0.726 °Brix	0.755 °Brix
pH	MSC	0.150	0.146
	Norm	0.127	0.124
	SG	0.119	0.110

**Table 5:** Predictive results of sugar and pH for 1D CNN tested with samples of TN and TB varieties.

Parameter	TN	TB
	RMSEP	RMSEP
Sugar	1.025 °Brix	1.203 °Brix
pH	0.234	0.158

**Table 6:** Optimized hyperparameters of 1D CNN for training process with samples of TF from 2012 until 2017 using BOGP.

Preprocessing	#Filters 1	Kernel Size 1	#Filters 2	Kernel Size 2	Neurons	Dropout 1/2	LR	Batch Size
SG	15	32	29	19	90	0.41/0.20	0.043	8

**Table 7:** Results obtained by the 1D CNN TF Model (2012–2017).

Parameter	RMSEV	RMSEP
Sugar	1.227 °Brix	1.396 °Brix
pH	0.182	0.223

## Appendix A

**Table A1:** Comparison of results from the present work and from other works published in the literature for the prediction of sugar content and pH using spectroscopic techniques in reflectance mode.

				RMSE	
Present Work Results:				Sugar (°Brix)	pH
	Different vintages (six)			0.755	0.110
	Testing with a different vintage			1.085	0.183
	Testing with different varieties			1.025/1.203	0.234/0.158
**Published works that**			**Algorithm**		
*Used a small number of berries per sample and* (as in the present work)	Used more than two vintages	[8]	SVM	1.411 worse than the present results	0.144 worse than the present results
	Tested with a different vintage	[3]	ANN	-	0.191 similar to the present results
		[4]	PLS	1.344 worse than the present results	-
			ANN	1.355 worse than the present results	-
	Tested with different varieties	[3]	ANN	-	0.170/0.176 similar to the present results
		[8]	SVM	2.443/3.186 worse than the present results	0.303/0.253 worse than the present results
	Used one vintage	[1]	PLS	1.270 worse than the present results	-
		[4]	PLS	0.939 worse than the present results	
			ANN	0.955 worse than the present results	
		[11]	PLS	1.150 worse than the present results	-
		[15]	ANN	0.950 worse than the present results	0.180 worse than the present results
	Used one vintage + blending varieties	[2]	LS-SVM *	0.960 worse than the present results	Different range of pH values
			PLS	0.930 worse than the present results	
*Used homogenate samples and*	Used more than two vintages	[5]	MPLS **	1.000 worse than the present results	0.120 similar to the present results
	Used one vintage	[7]	MPLS **	1.370 worse than the present results	0.120 similar to the present results
		[10]	MPLS **	-	0.150 worse than the present results
	Tested with a different vintage	[12,13]	PLS	1.090 similar to the present results	0.060 better than the present results
	Used one vintage + blending varieties	[12,13]	PLS	0.650 similar to the present results	0.050 better than the present results

* Least-squares support vector machines; ** modified partial least squares.
